# Dose-response relationship between dietary magnesium intake, serum magnesium concentration and risk of hypertension: a systematic review and meta-analysis of prospective cohort studies

**DOI:** 10.1186/s12937-017-0247-4

**Published:** 2017-05-05

**Authors:** Hedong Han, Xin Fang, Xin Wei, Yuzhou Liu, Zhicao Jin, Qi Chen, Zhongjie Fan, Jan Aaseth, Ayako Hiyoshi, Jia He, Yang Cao

**Affiliations:** 1 Department of Health Statistics, Second Military Medical University, No. 800 Xiangyin Road, Shanghai, 200433 China; 2 Unit of Biostatistics, Institute of Environmental Medicine, Karolinska Institutet, Nobelsväg 13, Box 210, Stockholm, 17 177 Sweden; 3 Mount Sinai St. Luke’s and West Medical Center, 1111 Amsterdam Ave., New York, NY 10025 USA; 4 Department of Cardiology, Peking Union Medical College Hospital, Peking Union Medical College, Chinese Academy of Medical Sciences, Beijing, 100730 China; 5 Faculty of Public Health, Hedmark University of Applied Sciences, 2411 Elverum, Norway; 6 Innlandet Hospital Trust, Kongsvinger Hospital Division, 2226 Kongsvinger, Norway; 7 Clinical Epidemiology and Biostatistics, School of Medical Sciences, Örebro University, 701 82 Örebro, Sweden

**Keywords:** Magnesium, Dietary intake, Serum concentration, Hypertension, Prospective cohort study, Dose-response relationship

## Abstract

**Background:**

The findings of prospective cohort studies are inconsistent regarding the association between dietary magnesium intake and serum magnesium concentration and the risk of hypertension. We aimed to review the evidence from prospective cohort studies and perform a dose-response meta-analysis to investigate the relationship between dietary magnesium intake and serum magnesium concentrations and the risk of hypertension.

**Methods:**

We searched systematically PubMed, EMBASE and the Cochrane Library databases from October 1951 through June 2016. Prospective cohort studies reporting effect estimates with 95% confidence intervals (CIs) for hypertension in more than two categories of dietary magnesium intake and/or serum magnesium concentrations were included. Random-effects models were used to combine the estimated effects.

**Results:**

Nine articles (six on dietary magnesium intake, two on serum magnesium concentration and one on both) of ten cohort studies, including 20,119 cases of hypertension and 180,566 participates, were eligible for inclusion in the meta-analysis. We found an inverse association between dietary magnesium intake and the risk of hypertension [relative risk (RR) = 0.92; 95% CI: 0.86, 0.98] comparing the highest intake group with the lowest. A 100 mg/day increment in magnesium intake was associated with a 5% reduction in the risk of hypertension (RR = 0.95; 95% CI: 0.90, 1.00). The association of serum magnesium concentration with the risk of hypertension was marginally significant (RR = 0.91; 95% CI: 0.80, 1.02).

**Conclusions:**

Current evidence supports the inverse dose-response relationship between dietary magnesium intake and the risk of hypertension. However, the evidence about the relationship between serum magnesium concentration and hypertension is limited.

**Electronic supplementary material:**

The online version of this article (doi:10.1186/s12937-017-0247-4) contains supplementary material, which is available to authorized users.

## Background

Cardiovascular diseases (CVD), stroke, and renal failure are the major causes of death with hypertension being the predominant risk factor [1]. The prevalence of hypertension has increased rapidly, with an estimated incidence of 29.6% among the U.S. adults [2]. Of the risk factors that increase mortality, high blood pressure is the leading factor in women and the second-leading factor in men [3]. The increasing incidence of hypertension has raised concerns on the potential risk factors of hypertension around the world [4]. Causes of hypertension include, but are not limited to, smoking, sedentary lifestyle, a diet rich in sodium, and an inadequate dietary intake of other mineral nutrients, such as, potassium, calcium and magnesium [5, 6].

Magnesium, the fourth most abundant mineral nutrient in human body, plays an essential role in regulating blood pressure, insulin metabolism, cardiac excitability and adenosine triphosphate (ATP) metabolism. However,the daily magnesium intake in western countries has been declining from approximately 500 mg/day to nearly 175–225 mg/day since the beginning of the last century, resulting in the increased likelihood of magnesium deficiencies among the western population [7]. However, the association between magnesium and blood pressure remained inconclusive, with the inverse association having been found in some studies [8–13], but not in others [14–16]. The effect of magnesium supplementation on blood pressure has been investigated in clinical trial since the 1980s [17]. Several studies support the role of magnesium deficiency caused by lack of dietary and/or supplementary magnesium intake in the development of hypertension [18–20]. Nevertheless, to date four meta-analyses have been conducted but reported inconsistent findings on the relationship between magnesium supplementation and blood pressure [21–24]. Also, the association between dietary magnesium intake and serum magnesium concentration and the risk of hypertension, studies have yielded inconsistent findings [25–33]. A negative association was demonstrated in two studies [25, 30], while others did not find any association [26–29, 31–33].

To our knowledge, the association between dietary magnesium intake and/or serum magnesium concentration and the risk of hypertension focusing prospectively collected data have not been summarized. Thus we conducted a dose-response meta-analysis to quantitatively elucidate the association between dietary magnesium intake and serum magnesium concentration and the risk of hypertension.

## Methods

### Data sources and search strategy

We conducted a systematic review for the prospective cohort studies that evaluated the association of dietary magnesium intake and/or serum magnesium concentration with the risk of hypertension. Retrospective cohort studies or historical cohort studies were not included because in such study design information of confounding factors were not always sufficiently available as the data were not collected for studying magnesium and blood pressure. We followed the standard criteria PRISMA (Preferred Reporting Items for Systematic Reviews and Meta-Analysis) and MOOSE (Meta-analysis of Observational Studies in Epidemiology) [34, 35]. The review was registered in the PROSPERO-international prospective register of systematic reviews (http://www.crd.york.ac.uk/prospero/, registration number: CRD42016039061). Prospective cohort studies published before June 2016 examining the relationship between dietary magnesium intake and/or serum magnesium concentration and the risk of hypertension were included in our study, regardless of language and publication status (published, in press or in progress). Two authors (HH and XW) independently searched PubMed, EMBASE, and the Cochrane Library electronic databases for articles using the following keywords: magnesium, Mg, hypertension, high blood pressure, combined with cohort, nested case-control or prospective. Furthermore, we manually searched potential articles in the references cited in relevant original and review articles.

### Selection criteria

Only studies that met the inclusion criteria were included in the meta-analysis: 1) the use of a prospective study design (cohort or nested case-control study); 2) dietary magnesium intake and/or serum magnesium concentration classified into three categories or more; 3) the outcome being the incident hypertension; 4) the use of adult population (age > 18 years); 5) the relative risk (RR), odds ratio (OR) or hazard ratio(HR)with corresponding 95% confidence interval (CI) being reported. We excluded cross-sectional studies because it may be difficult to ascertain the temporal association.

### Data extraction and quality assessment

Data extraction and quality assessment were conducted independently by two reviews (HH and XF). The following data were extracted from each eligible study: first author’s surname, year of publication, study country, study design, population, duration of follow-up, sample size (number of hypertension cases and total number of participants), sex, age, recruitment time, measure and range of exposure, outcome assessment, effect size (OR, RR or HR), 95% CIs and covariates adjusted in statistical analysis. For studies that reported several multivariable-adjusted effect estimates, we selected the one that was adjusted for all available potential confounding variables. Quality assessment for studies was conducted using the 9-star New-castle-Ottawa Scale (NOS) [36]. Studies with an NOS score of ≥ 7 were considered high-quality.

### Statistical analysis

RRs were used as the common measure of association and HRs and ORs were transformed into RRs [37, 38]. If a study reported results involving different populations (male and female) but did not report the overall results, the results for each population were regarded as a different study [39]; likewise, a study that reported both dietary magnesium intake and serum magnesium concentration was regarded as two separated studies. The statistical heterogeneity among the studies was assessed using I^2^ statistics [40].

A fixed-effect model was used to estimate the pooled RR when there was no evidence of heterogeneity; otherwise, a random-effect model was conducted [41]. Forest plots were produced to assess the RR estimates and corresponding 95% CI. Firstly, we pooled the risk estimates for the highest does/concentration categories of dietary/serum magnesium compared with the lowest categories. We subsequently transformed category-specific risk estimates into RRs associated with every 100 mg/day increment in dietary magnesium intake via the generalized least squares for trend estimation. Meanwhile, we also performed a two-stage random-effect dose-response meta-analysis to examine the potential nonlinear relationship between dietary magnesium intake and serum magnesium concentration and the risk of hypertension. Magnesium intake was modeled using restricted cubic splines with four knots at the 5th, 35th, 65th and 95th percentiles of the distribution [42, 43]. This method requires the effect estimate with its variance estimate for at least three known quantitative categories of exposure with one category serving as the common referent group. If a study did not report the distribution of magnesium intake for individuals without hypertension, we estimated the distribution using the method described by Aune et al. (for detailed description, see Additional file 1: Online supplement 1) [44]. For studies that did not set the lowest exposure as reference, we used the method described by Hamling et al. to make a transformation (for detailed calculation, see Additional file 2: Online supplement 2) [45]. The assigned dose of dietary magnesium intake for each category was the midpoint or median, and for the open categories half the width of the adjacent category was used.

Subgroup analyses were conducted for duration of follow-up, location, gender and adjustment for calcium, sodium, potassium, fiber, exercise, cholesterol, saturated fat intake and smoking. We also performed sensitivity analyses by excluding different studies. The possibility of publication bias was assessed by the combination of the Egger’s test, the Begg’s test and visual inspection of funnel plot [46].

Statistical software Stata (version 12.0, Stata Corp., College Station, TX) and Excel macro were used for all analyses. All statistical tests were two-sided. A p-value < 0.05 was considered to be statistically significant, except where otherwise specified.

## Results

### Description of the selected studies

**Fig. 1:**
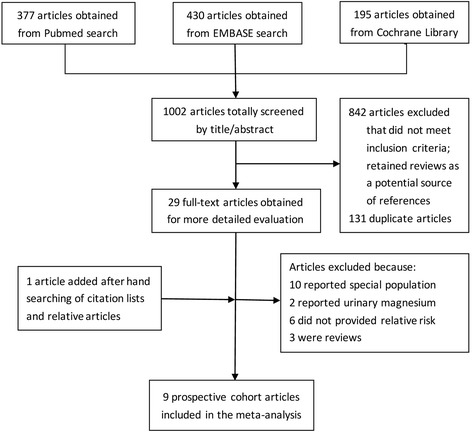
Screening and selection of articles on dietary magnesium intake, serum magnesium concentration and risk of hypertension

By June 2016, 1,002 studies were retrieved, of which 973 were excluded after review of title or abstract (Fig. 1), and 29 full-text articles were reviewed. We excluded further ten studies that focused on special populations of: pregnant women (six studies), children (one study), patients in the first year after renal transplantation (one study), patients with an implantable cardioverter defibrillator (one study) and patients with anesthesia (one study)). Additional two studies reporting the association between urinary magnesium and hypertension risk were also excluded. We further excluded three reviews and six articles that did not provide risk estimates. Thus, the meta-analysis included nine articles of ten independent prospective cohort studies [25–33], with a total of 20,119 hypertension cases and 180,566 study participants. The characteristics of the studies included in the analysis are summarized in Table 1. Among the ten cohort studies, eight were conducted in the USA [25–31], one in Mexico [33] and one in Netherlands [32]. Both male and female participants were recruited in six studies [28, 29, 31–33], whereas only males [26] or females [25, 27, 30] were recruited in some studies. Dietary magnesium intake was reported by seven studies [25–27, 29, 30, 33], while serum magnesium concentrations were examined in two studies [31, 32], and one study reported both [28]. RRs and corresponding 95% CIs for every magnesium category available from these studies were presented in Additional files 3 and 4: Tables S1 and S2. The range of dietary magnesium intake was 96–425 mg/day and serum magnesium levels were 0.66–0.95 mmol/L. The average score for quality of studies was 7 out of 9 in our assessment (Additional file 5: Table S3).

**Table 1:** Characteristic of the included studies with regard to magnesium and risk of hypertension

Source	Study	Case/Sample size	Gender, age range (years)	Follow-up (years)	Exposure assessment	Outcome ascertainment	Recruitment time	Hypertension definition (SBP/DBP mmHg)	Adjustments	Study quality
Witteman [25] 1989,USA	NHS	3275/58218	Female, 34–59	4	Validated 61-item FFQ	Validated self-reported	1980–1984	≥140/90	Age, Quetelet’s index, energy intake, alcohol use, calcium, magnesium, potassium, and fiber intake.	7
Ascherio [26], 1992,USA	HPFS	1248/30681	Male, 40–75	4	Validated 131-item FFQ	Validated self-reported	1986	≥140/90	Age, Quetelet’s index, alcohol use, magnesium, potassium, fiber, and energy.	6
Ascherio [27], 1996,USA	NHS	2526/41541	Female, 38–63	4	Validated 126-item and 61 item FFQ	Validated self-reported	1984–1988	≥140/90	Age, BMI, alcohol use, and energy.	7
Peacock [28], 1999,USA	ARIC	1597/7731	Female/Male, 45–64	6	61 item FFQ	Self-reported/AHTs treatment	1987–1989	≥140/90	Age, race, field center,BMI, WHR, diabetes, education, family history of hypertension, leisure activity score, hormone replacement therapy (women),baseline SBP and energy intake.	7
Song [30], 2006,USA	WHS	8544/28349	Female, ≥45	9.8	131-item FFQ	Validated self-reported/AHTs treatment/physician diagnosis	1992–1993	≥140/90	Age, treatment, family history of MI before 60, exercise, alcohol use, postmenopausal hormone use, multivitamin use, smoking, energy, BMI, history of diabetes, cholesterol, saturated fat intake, glycemic load, sodium intake.	7
He [29] 2006,USA	CARDIA	932/4637	Female/Male,18–30	15	Validated FFQ	Self-reported	1985–1986	≥130/85	Age, gender, race, education, smoking, physical activity, family history of diabetes, alcohol use, BMI, and intake of fiber, polyunsaturated fat, saturated fat, carbohydrates, and energy.	8
Khan [31], 2010, USA	FHS	551/2520	Female/Male middle-age	8	Standard colorimetrc assay	Physician diagnosis/AHTs treatment	1979–1982	≥140/90	Age,sex,BMI,diabetes,SBP,total/HDL cholesterol ratio,smoking,hemoglobin, albumin,GFR,calcium,potassium.	8
Joosten [32], 2013, Netherlands	PREVEND	1172/5511	Female/Male,28–75	7.6	Roche diagnostics	Self-reported/AHTs treatment	1997–1998	≥140/90	Age, sex, BMI, smoking, parental history of hypertension, alcohol intake, study design, and plasma levels of sodium, potassium, and calcium.	8
Huitrón [33], 2015, Mexico	HWCS	274/1378	Female/Male,20–87	7	Validated 116-item FFQ	Self-reported/physician diagnosis	2004–2006	≥140/90	Age, sex, time, physical activity, use of postmenopausal hormone, multivitamin use and alcohol, smoking, intake of saturated fat intake, cholesterol, sodium, and energy glycemic load,, history of diabetes, BMI and family history of hypertension.	7

Abbreviations: *NHS* Nurse’s Health Study, *HPFS* Health Professionals Follow-up Study, *ARIC* Atherosclerosis Risk in Communities Study, *WHS* Women’s Health Study, *CARDIA* Coronary Artery Risk Development in Young Adults, *HWCS* Health Workers Cohort Study, *FHS* Framingham Heart Study, *PREVEND* Prevention of Renal and Vascular End-Stage Disease study, *FFQ* food-frequency questionnaire, *BMI* body mass index, *MI* myocardial infarction, *SBP* systolic blood pressure, *WHR* waist-to-hip ratio, *HDL* high density lipoprotein, *GFR* glomerular filtration rate, *AHTs* antihypertensive medications

### Dietary magnesium intake and risk of hypertension

**Fig. 2:**
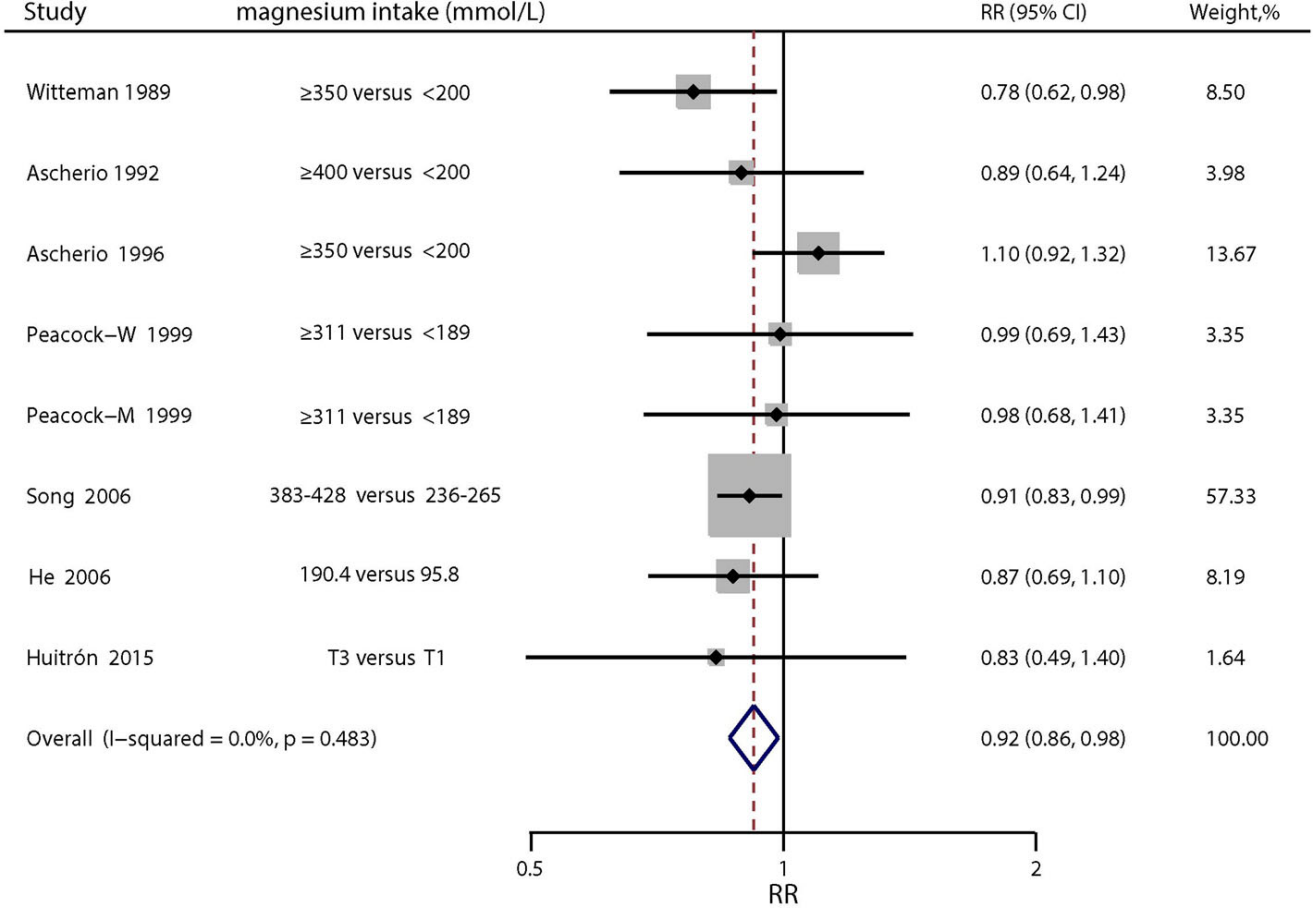
Forest plots of relative risks of hypertension for the highest versus lowest categories of dietary magnesium intake

**Fig. 3:**
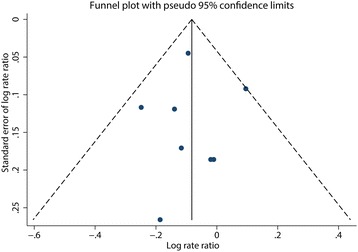
Funnel plot of studies reporting dietary magnesium intake and the risk of hypertension

**Fig. 4:**
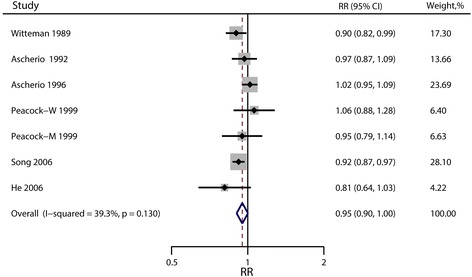
Forest plots of relative risks of hypertension for an increment of 100 mg/d of dietary magnesium intake

**Fig. 5:**
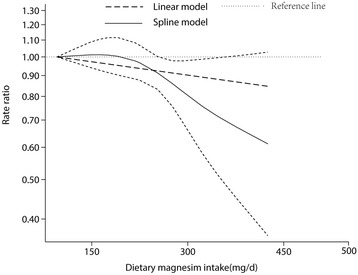
Dose-response relationship between dietary magnesium intake and risk of hypertension

Multivariable-adjusted relative risks for the highest vs. lowest categories of individual studies and all studies combined are shown in Fig. 2. The summary result indicated a statistically significant inverse relationship between dietary magnesium intake and hypertension risk (pooled RR = 0.92; 95% CI: 0.86, 0.98), and there was no evidence of heterogeneity (I^2^ = 0; *p* = 0.48). The funnel plot showed reasonable symmetry, with no evidence of publication bias (Fig. 3, Egger’s test *p* = 0.95 and Begg’s test *p* = 0.71). Figure 4 shows the individual and combined RRs and corresponding 95% CIs for hypertension risk of per 100 mg/day increment in dietary magnesium intake (pooled RR = 0.95; 95% CI: 0.90, 1.00; I^2^ = 39.3%; *p* = 0.13). The dose-response meta-analysis suggested a marginal linear relationship between dietary magnesium intake and hypertension risk (Fig. 5, p for linearity = 0.057), and we did not find evidence of a nonlinear relationship (Fig. 5, p for non-linearity = 0.21).

### Serum magnesium concentration and risk of hypertension

**Fig. 6:**
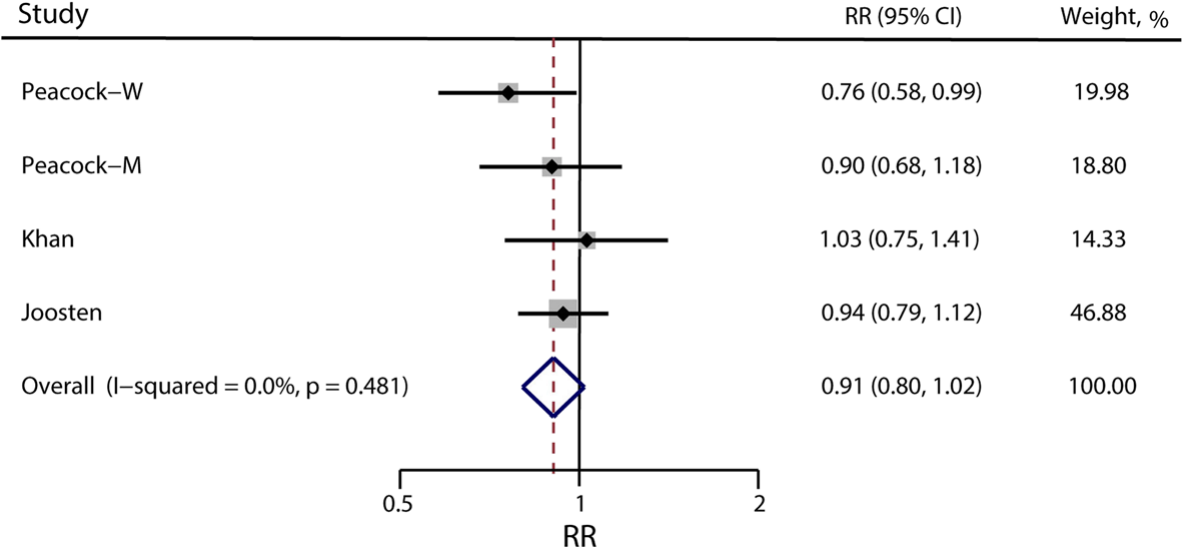
Forest plots of relative risks of hypertension for the highest versus lowest categories of serum magnesium concentration

Four prospective cohort studies were included in the analysis. There was no indication of publication bias (Egger’s test *p* = 0.86 and Begg’s test *p* = 0.73). The pooled RR is 0.91 (95% CI: 0.80, 1.02; *p* = 0.10) for the highest vs. lowest categories (Fig. 6), which is marginally significant, and no evidence of heterogeneity was observed (I^2^ = 0; *p* = 0.48). The dose-response meta-analysis demonstrated no association between the risk of hypertension and per-unit increment of serum magnesium concentration (p for linearity = 0.23). We did not find the evidence of a nonlinear relationship between serum magnesium concentration and risk of hypertension either (p for non-linearity = 0.91). The results were hardly changed in sensitivity analysis when different studies were excluded.

### Subgroup analysis

There was no statistically significant heterogeneity in the effect of dietary magnesium intake on hypertension risk in different populations. In the studies conducted in the USA, the hypertension risk associated with lower dietary magnesium intake tended to be lower, with duration of follow-up more than 8 years and adjusting for calcium, sodium, potassium, fiber, exercise, cholesterol, saturated fat intake or smoking, (Table 2). The reduced hypertension risk associated with 100 mg/day was tended to be observed when the duration of follow-up was more than 8 years and when the results were adjusted separately for calcium, sodium, fiber, cholesterol, saturated fat intake or smoking (Table 2). Because of the limited data availability, subgroup analysis was not conducted for serum magnesium concentrations.

**Table 2:** Pooled relative risk (RR) of hypertension and RR per 100 mg/d increment in dietary magnesium intake

	Highest versus Lowest dietary magnesium intake				Per 100 mg/day increment in dietary magnesium intake			
	Number of studies.^a^	RR(95%CI)	I^2^ (%)	p for heterogeneity	Number of studies. ^a^	RR(95%CI)	I^2^ (%)	p for heterogeneity
All studies	8	0.92(0.86,0.98)	0	0.48	7	0.95(0.90,1.00)	39.30	0.13
Location								
USA	7	0.92(0.86,0.99)	5.40	0.39	7	0.95(0.90,1.00)	39.30	0.13
Other area	1	0.83(0.49,1.39)	-	-	0	-	-	-
Gender								
Male	2	0.93(0.73,1.19)	0	0.70	2	0.81(0.64,1.03)	0	0.85
Female	4	0.94(0.82,1.07)	49.40	0.12	4	0.96(0.89,1.03)	62.60	0.05
Both^b^	2	0.86(0.70,1.07)	0	0.87	1	0.96(0.88,1.06)	-	-
Follow-up (year)								
≥ 8	2	0.90(0.83,0.98)	0	0.72	2	0.91(0.86,0.97)	4.40	0.31
< 8	6	0.94(0.83,1.07)	14.70	0.32	5	0.98(0.92,1.03)	23.90	0.26
Adjusted alcohol								
Yes	6	0.92(0.84,1.00)	19.60	0.29	5	0.94(0.89,1.00)	53.00	0.07
No	2	0.98(0.76,1.27)	0	0.97	2	1.00(0.88,1.14)	0	0.41
Adjusted calcium								
Yes	1	0.78(0.62,0.98)	-	-	1	0.90(0.82,0.99)	-	-
No	7	0.94(0.87,1.00)	0	0.64	6	0.96(0.91,1.02)	40.50	0.14
Adjusted sodium								
Yes	2	0.91(0.83,0.99)	0	0.73	1	0.92(0.87,0.97)	-	-
No	6	0.93(0.83,1.05)	18.30	0.30	6	0.96(0.90,1.03)	34.10	0.18
Adjusted potassium								
Yes	2	0.81(0.67,0.98)	0	0.52	2	0.93(0.86,1.00)	0	0.32
No	6	0.94(0.87,1.01)	0	0.52	5	0.96(0.89,1.03)	52.20	0.08
Adjusted fiber								
Yes	3	0.84(0.72,0.97)	0	0.74	3	0.92(0.85,0.99)	6.20	0.35
No	5	0.94(0.88,1.02)	0	0.44	4	0.97(0.91,1.05)	53.30	0.09
Adjusted diabetes								
Yes	2	0.91(0.84,1.00)	0	0.66	2	0.96(0.85,1.09)	50.60	0.16
No	6	0.92(0.82,1.04)	19.80	0.28	5	0.95(0.89,1.02)	40.40	0.15
Adjusted family history of hypertension								
Yes	2	0.93(0.69,1.26)	0	0.59	1	1.06(0.88,1.28)	-	-
No	6	0.92(0.84,1.01)	19.30	0.29	6	0.94(0.90,1.00)	41.30	0.13
Adjusted education								
Yes	2	0.90(0.74,1.10)	0	0.56	2	0.94(0.72,1.22)	67.00	0.08
No	6	0.93(0.84,1.02)	18.20	0.30	5	0.95(0.90,1.00)	41.50	0.15
Adjusted exercise								
Yes	4	0.91(0.84,0.98)	0	0.93	3	0.93(0.84,1.03)	38.70	0.20
No	4	0.94(0.78,1.12)	45.90	0.14	4	0.97(0.91,1.03)	33.50	0.21
Adjusted cholesterol								
Yes	3	0.90(0.83,0.98)	0	0.89	2	0.91(0.86,0.97)	4.40	0.31
No	5	0.95(0.82,1.10)	28.30	0.23	5	0.98(0.92,1.03)	23.90	0.26
Adjusted saturated fat intake								
Yes	3	0.90(0.83,0.98)	0	0.89	2	0.91(0.86,0.97)	4.40	0.31
No	5	0.95(0.82,1.10)	28.30	0.23	5	0.98(0.92,1.03)	23.90	0.26
Adjusted smoking								
Yes	3	0.90(0.83,0.98)	0	0.89	2	0.91(0.86,0.97)	4.40	0.31
No	5	0.95(0.82,1.10)	28.30	0.23	5	0.98(0.92,1.03)	23.90	0.26

^a^If a study reported results involving different populations (male and female) but did not report the overall results, the results for each population were regarded as different studies

^b^“Both” means that the original study did not report effect estimates by gender separately

## Discussion

Magnesium regulates the physical properties of cellular membranes and their permeability, and could therefore alter the permeability of cells for calcium and sodium, which are important mechanisms in the development of hypertension [47]. It has also been suggested that magnesium acts as a calcium antagonist that modulates vascular smooth muscle tone and contractibility by affecting calcium ion concentrations, causing vasorelaxation [5]. Thus, magnesium deficiency might affect blood pressure values, leading to hypertension, and supra-nutritional magnesium intake might act as a mild antihypertensive agent.

The present systematic review and meta-analysis of prospective cohort studies demonstrated a statistically significant inverse association between dietary magnesium intake and the risk of hypertension. For a 100 mg/day increase in dietary magnesium intake, the risk decreased by 5%. However, there was no significant association between serum magnesium concentration and the risk of hypertension. The effect estimates show no heterogeneity among studies included for both dietary magnesium intake and serum magnesium concentration, which means that our combination of former studies is relatively reliable.

Meanwhile, results indicated that other factors may be associated with the findings in the studies, such as duration of follow-up and measures adjusted for, such as calcium, sodium, potassium, fiber, exercise, cholesterol, saturated fat intake and smoking, and the combination of several ions supplementation [48]. In our meta-analysis, subgroup analysis showed that after adjusting separately for these factors, the inverse association presents a little lower than that without adjustment (Table 2).

### Dietary magnesium and blood pressure

For the past decades, a number of studies have examined the relationship between magnesium intake and blood pressure. Four meta-analyses [21–24] and two systematic reviews have been conducted [49, 50], but results remained inconsistent. Mizushima et al. [50] found a significantly negative association between dietary magnesium intake and blood pressure, however, no quantitative results were produced because of the difficulty in combining estimates due to methodological differences in dietary data collection, study design and potential biases in studies involved. The present study effectively avoided these problems and made a quantitative assessment of the effect of dietary magnesium intake on hypertension. On the other hand, Burgess et al. [49] reviewed relevant epidemiologic studies and concluded that higher dietary magnesium intake was not associated. Of the four meta-analyses of randomized controlled trials (RCTs) regarding the effect of magnesium supplementation on the subsequent blood pressure, Jee et al. reported a small and non-significant reduction in blood pressure by analyzing 20 studies of hypertensive and normotensive individuals. Dickinson et al. reported a weak benefit of magnesium supplementation on blood pressure. A meta-analysis in 2012 involving 22 RCTs found a reduction of 3–4 mmHg SBP and 2–3 mmHg DBP with oral magnesium supplementation. Notably, a significant reduction of 18.7 mmHg in SBP and 10.9 mmHg in DBP was reported by Rosanoff et al., but among a specific population of SBP > 155mmhg. For normotensive individuals, studies showed that lower dietary magnesium intake may correlate with elevated blood pressure and thus with the development of hypertension in general population [51, 52].

### Serum magnesium and blood pressure

Studies reporting the association between serum magnesium and blood pressure were limited. Shibutani et al. reported that Japanese children who had a hereditary predisposition to hypertension tended to have higher blood pressure and lower erythrocyte magnesium levels, rather than serum magnesium [53]. Rinner et al. found no relationship between serum magnesium and blood pressure [54]. But Guerrero-Romero et al. conducted a study using healthy Mexican children and found that serum magnesium level <1.8 mg/dL was significantly associated with prehypertension and hypertension [55]. Compared with dietary magnesium, serum magnesium may affect blood pressure more directly. Therefore, further research is needed to examine the relationship between serum magnesium levels and blood pressure, the incidence of hypertension and other cardiovascular diseases.

### Quality of included studies

The risk of bias in the studies included was low (Egger’s test *p* = 0.95 and Begg’s test *p* = 0.71 for dietary magnesium intake and Egger’s test *p* = 0.86 and Begg’s test *p* = 0.73 for serum magnesium concentration), and studies were generally considered of high quality (with relatively high NOS scores). The degree of follow-up remained limited, although in some studies only subgroups were followed for longer duration. Differential loss to follow-up between dose categories did not appear to be an issue, and neither did selective publication. However, eight of 10 studies included were from the USA, which may have affected generalizability. Although difference between the USA and non-USA reports indicated possible variations between different populations, evidence from other regions has been limited, and further studies are necessary to assess this issue.

### Strengths and limitations

To the best of our knowledge, this is the first meta-analysis focusing on prospective cohort studies to examine the association of dietary magnesium intake and serum magnesium concentration with the risk of hypertension with including the assessment of both linear and nonlinear dose-response relationships.

A strength of our meta-analysis is the prospective feature of the studies included, which reduced risk of selection and recall biases that could be of concern in retrospective case-control studies. As a method for quantifying nutrient intakes, FFQ was used in a case-control study, while the reliability of FFQ may be low even though the majority of FFQs have been validated before application [56]. Cross-sectional studies suggested no significant benefit of dietary magnesium intake on hypertension risk in normotensive individuals, which was different from our result [33, 57]. These studies, however, used the 24-h dietary recall method to assess magnesium exposure, which may be vulnerable to recall and reporting biases, resulting in the misclassification of dietary intake. A study assessed dietary magnesium intake with an FFQ only at baseline, but this may result in underestimation the relative risk [58]. Furthermore, most studies included in our study were adjusted for potential confounders such as alcohol consumption, calcium, sodium, potassium, fiber, physical activity, cholesterol, saturated fat intake and smoking, removing effect of these measures.

However, there are also limitations in our study. First, residual confounding cannot be ruled out in observational studies. For example, only one study was adjusted for calcium intake, which might affect the absorption of magnesium [59]. In addition, unmeasured or unknown factors might also be a source of confounding. Second, the range of dietary magnesium levels is centered at approximately 150 mg/day to 400 mg/day that may weaken the dose-response relationship at higher levels of magnesium intake. In Fig. 5, when the intake of dietary magnesium is beyond 370 mg/day, the insignificant result may be caused by lack of statistical power. Third, there may be specific subgroups that show different associations. For example, Choi et al. reported obese women may benefit more with increased magnesium intake [51]. In our study, however, only a small number of studies were included after applying the selection criteria and it was not possible to assess associations in specific subgroups. Four, the studies included did not report the effect of supplemental magnesium on hypertension risk separately from other form of magnesium intakes. Thus, we could not investigate the effect of supplemental magnesium specifically, but the use of supplemental magnesium was relatively minor compared with dietary magnesium, as indicated by the study conducted by He et al. that dietary magnesium intake was nearly comparable to total magnesium [29]. Finally, we only had four studies reporting the serum magnesium concentration and hypertension risk and failed to find any statistically significant association with the risk of hypertension (RR = 0.91; 95% CI: 0.80, 1.02).

### Inferences

Magnesium is mainly consumed through diet, and low magnesium consumption is common worldwide. It has been estimated that magnesium intake in a normal Western diet is often inadequate for the body’s needs; In the Unit States, 56% of women and 53% of men consume insufficient amounts of magnesium [60]. For people aged more than 30, the recommended dietary allowance of magnesium was 350 mg/day and 420 mg/day for men and women, respectively [61]. On the basis of the studies we have reviewed, current evidence from population-based prospective cohort studies support the recommendation for increasing the dietary magnesium intake.

### Implication for future research

More evidence is needed, especially on the dose-response relationship between dietary magnesium intake and hypertension in populations in Asia, Africa and Europe. Further research into biological mechanism and how low magnesium intake could be reduced will help developing evidence-based dietary recommendations and interventions.

## Conclusions

This meta-analysis including 10 prospective cohort studies found that magnesium intake showed a reduced risk of incidence of hypertension with no evidence of heterogeneity. Our findings suggest that increased dietary magnesium intake was associated with lower risk of hypertension in a linear dose-response pattern. Magnesium consumption have often found inadequate and low among adult population worldwide, and our results support the recommendation for increasing the dietary magnesium intake. However, no statistically significant association was found between serum magnesium concentration and risk of hypertension. The data for the high level of dietary magnesium intake were sparse, and further research including randomized clinical trials is needed to examine the effects of dietary magnesium intake on hypertension at high doses.

## Additional files

Additional file 1: Online supplement 1. Method for estimation of distribution of person-years. (DOC 22 kb)

Additional file 2: Online supplement 2. (XLS 125 kb)

Additional file 3: Table S1. Dietary magnesium and hypertension risk by study and dose category. (DOC 64 kb)

Additional file 4: Table S2. Serum magnesium and hypertension risk by study and concentration category. (DOC 44 kb)

Additional file 5: Table S3. Quality assessment of included cohort studies. (DOCX 16 kb)

## Abbreviations

AHTs: Antihypertensive medications
ARIC: Atherosclerosis risk in communities study
BMI: Body mass index
CARDIA: Coronary Artery Risk Development in Young Adults
CI: Confidence interval
DBP: Diastolic blood pressure
FFQ: Food frequency questionnaire
FHS: Framingham heart study
GFR: Glomerular filtration rate
HDL: High density lipoprotein
HPFS: Health Professionals follow-up study
HR: Hazard ratio
HWCS: Health Workers Cohort Study
Mg: Magnesium
MI: Myocardial infarction
NHS: Nurse’s health study
NOS: New-castle-Ottawa
OR: Odds ratio
PREVEND: Prevention of renal and vascular end-stage disease study
RR: Relative risk
SBP: Systolic blood pressure
WHR: Waist-to-hip ratio
WHS: Women’s health study
