# Supplementary material for: Dose-response relationship between dietary magnesium intake, serum magnesium concentration and risk of hypertension: a systematic review and meta-analysis of prospective cohort studies
Source: Nutr J. 2017 May 5;16:26. doi: 10.1186/s12937-017-0247-4 (PMC5420140; doi:10.1186/s12937-017-0247-4)
Supplement: Supplementary file 1 — Online supplement 1. Method for estimation of distribution of person-years. (DOC 22 kb) [file 12937_2017_247_MOESM1_ESM.doc]

Additional file 1: Online supplement 1: Method for estimation of distribution of person-years

We estimated the distribution of cases and person-years based on the following method. Because the number of cases and person-years are only used as starting points for the iterations with the glst method, the exact number of cases or person-years is not needed. We used slightly different numbers of cases/person years in one study to check that this is correct and the results were identical.

The example is taken from the paper by Samanic paper (ref. no 6 in the article) based on the pancreatic cancer analysis (698 cases, total 6918667 person-years). The number of cases, but not person-years, was provided in this study.

BMI <25 25-29.9 30.0+

cases 352 289 57

RR 1.00 0.95 1.16

cases (if rr=1) 352 304 49

new total /705 /705 /705

proportion of new total 0.4992907801 0.4312056738 0.0695035461

x 6918667 x 6918667 x 6918667

person-years per category 3454426.644 2983368.465 480871.8908

First we calculate the distribution of cases in each category if the RR is 1.00 in each category by dividing the number of cases with the relative risk. Then we calculated the proportion of the total number of cases (if the RR=1) for each category by dividing with the new total (352+304+49=705=new total). Then we multiplied this proportion with the total number of person-years provided in the text (=6918667) and get the approximated number of person-years for each category.
