# Supplementary material for: Dose-response relationship between dietary magnesium intake, serum magnesium concentration and risk of hypertension: a systematic review and meta-analysis of prospective cohort studies
Source: Nutr J. 2017 May 5;16:26. doi: 10.1186/s12937-017-0247-4 (PMC5420140; doi:10.1186/s12937-017-0247-4)
Supplement: Supplementary file 3 — Dietary magnesium and hypertension risk by study and dose category. (DOC 64 kb) [file 12937_2017_247_MOESM3_ESM.doc]

**Table S1** Dietary magnesium and hypertension risk by study and dose category

| Study id | Author | Year | Study^1^ | Type | RR | Lb | Ub | Dose | Case | N |
| --- | --- | --- | --- | --- | --- | --- | --- | --- | --- | --- |
| 1 | Witteman | 1989 | CI | 3 | 1 | 1 | 1 | 175 | 287 | 4494 |
| 1 | Witteman | 1989 | CI | 3 | 0.91 | 0.77 | 1.09 | 225 | 695 | 12154 |
| 1 | Witteman | 1989 | CI | 3 | 0.93 | 0.77 | 1.12 | 275 | 971 | 16412 |
| 1 | Witteman | 1989 | CI | 3 | 0.85 | 0.69 | 1.04 | 325 | 731 | 13324 |
| 1 | Witteman | 1989 | CI | 3 | 0.78 | 0.62 | 0.98 | 375 | 591 | 11834 |
| 2 | Ascherio | 1992 | CI | 3 | 1 | 1 | 1 | 225 | 78 | 1507 |
| 2 | Ascherio | 1992 | CI | 3 | 0.96 | 0.66 | 1.39 | 275 | 201 | 4522 |
| 2 | Ascherio | 1992 | CI | 3 | 0.89 | 0.63 | 1.26 | 325 | 292 | 7317 |
| 2 | Ascherio | 1992 | CI | 3 | 0.94 | 0.67 | 1.31 | 375 | 299 | 7238 |
| 2 | Ascherio | 1992 | CI | 3 | 0.9 | 0.65 | 1.26 | 425 | 378 | 10097 |
| 3 | Ascherio | 1996 | IR | 2 | 1 | 1 | 1 | 175 | 182 | 12591 |
| 3 | Ascherio | 1996 | IR | 2 | 1.12 | 0.95 | 1.33 | 225 | 614 | 36312 |
| 3 | Ascherio | 1996 | IR | 2 | 1.09 | 0.92 | 1.28 | 275 | 765 | 45674 |
| 3 | Ascherio | 1996 | IR | 2 | 1.12 | 0.94 | 1.33 | 325 | 551 | 31180 |
| 3 | Ascherio | 1996 | IR | 2 | 1.1 | 0.92 | 1.32 | 375 | 414 | 23537 |
| 4 | Peacock-W | 1999 | CI | 3 | 1 | 1 | 1 | 162.5 | 232 | 1237 |
| 4 | Peacock-W | 1999 | CI | 3 | 0.98 | 0.76 | 1.25 | 217.5 | 202 | 1094 |
| 4 | Peacock-W | 1999 | CI | 3 | 1.21 | 0.91 | 1.59 | 277.5 | 232 | 1020 |
| 4 | Peacock-W | 1999 | CI | 3 | 0.99 | 0.69 | 1.43 | 342.5 | 156 | 839 |
| 5 | Peacock-M | 1999 | CI | 3 | 1 | 1 | 1 | 162.5 | 146 | 695 |
| 5 | Peacock-M | 1999 | CI | 3 | 1.13 | 0.85 | 1.49 | 217.5 | 198 | 838 |
| 5 | Peacock-M | 1999 | CI | 3 | 0.97 | 0.72 | 1.31 | 277.5 | 186 | 914 |
| 5 | Peacock-M | 1999 | CI | 3 | 0.98 | 0.68 | 1.41 | 342.5 | 225 | 1094 |
| 6 | Song | 2006 | CI | 3 | 1 | 1 | 1 | 253 | 1760 | 5668 |
| 6 | Song | 2006 | CI | 3 | 1 | 0.93 | 1.07 | 292 | 1739 | 5671 |
| 6 | Song | 2006 | CI | 3 | 1.02 | 0.95 | 1.1 | 320 | 1794 | 5669 |
| 6 | Song | 2006 | CI | 3 | 0.89 | 0.83 | 0.97 | 350 | 1618 | 5670 |
| 6 | Song | 2006 | CI | 3 | 0.91 | 0.83 | 0.99 | 400 | 1633 | 5671 |
| 7 | He | 2006 | CI | 3 | 1 | 1 | 1 | 95.8 | 293 | 1264 |
| 7 | He | 2006 | CI | 3 | 1 | 0.84 | 1.19 | 120.4 | 268 | 1156 |
| 7 | He | 2006 | CI | 3 | 0.82 | 0.67 | 1 | 147.1 | 199 | 1047 |
| 7 | He | 2006 | CI | 3 | 0.87 | 0.69 | 1.1 | 190.4 | 172 | 853 |

^1^If a study reported cumulative incidence, type was assigned with 3; If a study reported incidence rate, type was assigned with 2.
