# Supplementary material for: Dose-response relationship between dietary magnesium intake, serum magnesium concentration and risk of hypertension: a systematic review and meta-analysis of prospective cohort studies
Source: Nutr J. 2017 May 5;16:26. doi: 10.1186/s12937-017-0247-4 (PMC5420140; doi:10.1186/s12937-017-0247-4)
Supplement: Supplementary file 4 — Serum magnesium and hypertension risk by study and concentration category. (DOC 44 kb) [file 12937_2017_247_MOESM4_ESM.doc]

**Table S2**-- Serum magnesium and hypertension risk by study and concentration category

| id | Author | Year | Study^1^ | Type | RR | Lb | Ub | Concentration | Case | n |
| --- | --- | --- | --- | --- | --- | --- | --- | --- | --- | --- |
| 1 | Peacock-W | 1999 | CI | 3 | 1 | 1 | 1 | 0.78 | 208 | 922 |
| 1 | Peacock-W | 1999 | CI | 3 | 0.82 | 0.64 | 1.05 | 0.83 | 209 | 1131 |
| 1 | Peacock-W | 1999 | CI | 3 | 0.93 | 0.73 | 1.19 | 0.88 | 236 | 1127 |
| 1 | Peacock-W | 1999 | CI | 3 | 0.76 | 0.58 | 0.99 | 0.93 | 169 | 991 |
| 2 | Peacock-M | 1999 | CI | 3 | 1 | 1 | 1 | 0.78 | 161 | 697 |
| 2 | Peacock-M | 1999 | CI | 3 | 0.91 | 0.7 | 1.19 | 0.83 | 196 | 936 |
| 2 | Peacock-M | 1999 | CI | 3 | 0.92 | 0.71 | 1.19 | 0.88 | 214 | 1007 |
| 2 | Peacock-M | 1999 | CI | 3 | 0.9 | 0.68 | 1.18 | 0.93 | 184 | 888 |
| 3 | Khan | 2010 | CI | 3 | 1 | 1 | 1 | 0.66 | 139 | 630 |
| 3 | Khan | 2010 | CI | 3 | 0.97 | 0.72 | 1.31 | 0.79 | 135 | 630 |
| 3 | Khan | 2010 | CI | 3 | 0.96 | 0.7 | 1.32 | 0.83 | 134 | 630 |
| 3 | Khan | 2010 | CI | 3 | 1.03 | 0.75 | 1.41 | 0.95 | 143 | 630 |
| 4 | Joosten | 2013 | CI | 3 | 1 | 1 | 1 | 0.66 | 268 | 8317 |
| 4 | Joosten | 2013 | CI | 3 | 1 | 0.84 | 1.2 | 0.79 | 221 | 6990 |
| 4 | Joosten | 2013 | CI | 3 | 0.92 | 0.77 | 1.09 | 0.83 | 269 | 9580 |
| 4 | Joosten | 2013 | CI | 3 | 0.94 | 0.79 | 1.12 | 0.95 | 263 | 8773 |

^1^If a study reported cumulative incidence, type was assigned with 3; If a study reported incidence rate, type was assigned with 2.
