# Supplementary material for: Dose-response relationship between dietary magnesium intake, serum magnesium concentration and risk of hypertension: a systematic review and meta-analysis of prospective cohort studies
Source: Nutr J. 2017 May 5;16:26. doi: 10.1186/s12937-017-0247-4 (PMC5420140; doi:10.1186/s12937-017-0247-4)
Supplement: Supplementary file 5 — Quality assessment of included cohort studies. (DOCX 16 kb) [file 12937_2017_247_MOESM5_ESM.docx]

**Table S3**. Quality assessment of included cohort studies

| Author  (Publication Year) | Newcastle-Ottawa Scale | | | | | | | | | |
| --- | --- | --- | --- | --- | --- | --- | --- | --- | --- | --- |
|  | Selection | | | Comparability | | | Outcome | | | Total |
|  | a | b | c | d | e | f | g | h | i |  |
| Witteman 1989 | 0 | 1 | 1 | 1 | 1 | 1 | 1 | 0 | 1 | 7 |
| Ascherio 1992 | 0 | 1 | 1 | 1 | 1 | 1 | 0 | 0 | 1 | 6 |
| Ascherio 1996 | 0 | 1 | 1 | 1 | 1 | 1 | 1 | 0 | 1 | 7 |
| Peacock -W 1999 | 1 | 1 | 1 | 1 | 1 | 1 | 0 | 0 | 1 | 7 |
| Peacock -M 1999 | 1 | `1 | 1 | 1 | 1 | 1 | 0 | 0 | 1 | 7 |
| Song 2006 | 1 | 1 | 1 | 1 | 1 | 1 | 0 | 1 | 0 | 7 |
| He 2006 | 1 | 1 | 1 | 1 | 1 | 1 | 0 | 1 | 1 | 8 |
| Khan 2010 | 0 | 1 | 1 | 1 | 1 | 1 | 1 | 1 | 1 | 8 |
| Joosten 2013 | 1 | 1 | 1 | 1 | 1 | 1 | 1 | 1 | 0 | 8 |
| Huitrón 2015 | 0 | 1 | 1 | 1 | 1 | 1 | 0 | 1 | 1 | 7 |

1. Representativeness of the exposed cohort;
2. Selection of the non-exposed cohort.
3. Ascertainment of exposure.
4. Demonstration that outcome of interest was not present at start of study.
5. Comparability of cohorts on the basis of the design or analysis (adjusted for age).
6. Comparability of cohorts on the basis of the design or analysis (adjusted for any other factor).
7. Assessment of outcome.
8. Was follow-up long enough for outcomes to occur.
9. Adequacy of follow-up of cohorts.
